# Altered fetal growth, placental abnormalities, and stillbirth

**DOI:** 10.1371/journal.pone.0182874

**Published:** 2017-08-18

**Authors:** Radek Bukowski, Nellie I. Hansen, Halit Pinar, Marian Willinger, Uma M. Reddy, Corette B. Parker, Robert M. Silver, Donald J. Dudley, Barbara J. Stoll, George R. Saade, Matthew A. Koch, Carol Hogue, Michael W. Varner, Deborah L. Conway, Donald Coustan, Robert L. Goldenberg, for the Eunice Kennedy Shriver National Institute of Child Health and Human Development (NICHD) Stillbirth Collaborative Research Network (SCRN)

**Affiliations:** 1 The University of Texas at Austin Dell Medical School, Austin, Texas, United States of America; 2 RTI International, Research Triangle Park, North Carolina, United States of America; 3 Brown University School of Medicine, Providence, Rhode Island, United States of America; 4 The Pregnancy and Perinatology Branch, the *Eunice Kennedy Shriver* National Institute of Child Health and Human Development, National Institutes of Health, Bethesda, Maryland, United States of America; 5 University of Utah School of Medicine and Intermountain Health Care, Salt Lake City, Utah, United States of America; 6 University of Virginia School of Medicine, Charlottesville, Virginia, United States of America; 7 University of Texas Health Science Center Houston, Houston, Texas, United States of America; 8 University of Texas Medical Branch at Galveston, Galveston, Texas, United States of America; 9 Rollins School of Public Health, Emory University, Atlanta, Georgia, United States of America; 10 University of Texas Health Science Center at San Antonio, San Antonio, Texas, United States of America; 11 Columbia University Medical Center, New York, New York, United States of America; Stellenbosch University, SOUTH AFRICA

## Abstract

**Background:**

Worldwide, stillbirth is one of the leading causes of death. Altered fetal growth and placental abnormalities are the strongest and most prevalent known risk factors for stillbirth. The aim of this study was to identify patterns of association between placental abnormalities, fetal growth, and stillbirth.

**Methods and findings:**

Population-based case-control study of all stillbirths and a representative sample of live births in 59 hospitals in 5 geographic areas in the U.S. Fetal growth abnormalities were categorized as small (<10th percentile) and large (>90th percentile) for gestational age at death (stillbirth) or delivery (live birth) using a published algorithm. Placental examination by perinatal pathologists was performed using a standardized protocol. Data were weighted to account for the sampling design. Among 319 singleton stillbirths and 1119 singleton live births at ≥24 weeks at death or delivery respectively, 25 placental findings were investigated. Fifteen findings were significantly associated with stillbirth. Ten of the 15 were also associated with fetal growth abnormalities (single umbilical artery; velamentous insertion; terminal villous immaturity; retroplacental hematoma; parenchymal infarction; intraparenchymal thrombus; avascular villi; placental edema; placental weight; ratio birth weight/placental weight) while 5 of the 15 associated with stillbirth were not associated with fetal growth abnormalities (acute chorioamnionitis of placental membranes; acute chorioamionitis of chorionic plate; chorionic plate vascular degenerative changes; perivillous, intervillous fibrin, fibrinoid deposition; fetal vascular thrombi in the chorionic plate). Five patterns were observed: placental findings associated with (1) stillbirth but not fetal growth abnormalities; (2) fetal growth abnormalities in stillbirths only; (3) fetal growth abnormalities in live births only; (4) fetal growth abnormalities in stillbirths and live births in a similar manner; (5) a different pattern of fetal growth abnormalities in stillbirths and live births.

**Conclusions:**

The patterns of association between placental abnormalities, fetal growth, and stillbirth provide insights into the mechanism of impaired placental function and stillbirth. They also suggest implications for clinical care, especially for placental findings amenable to prenatal diagnosis using ultrasound that may be associated with term stillbirths.

## Introduction

Worldwide over 2.6 million pregnancies result in third trimester stillbirths annually [1]. In the US, about 1 in 160 pregnancies results in stillbirth [2]. The number of stillbirths occurring globally would place it as the fifth leading cause of death following ischemic heart disease, stroke, chronic obstructive pulmonary disease, and lower respiratory infections (WHO www.who.int/mediacentre/factsheets/fs310/en/). While the last decades have seen a steady improvement in neonatal and infant mortality, the rate of stillbirth has declined five times more slowly and exceeded the infant mortality rate in the U.S. in 2013 [2, 3]. Limited understanding of the mechanism and ability to predict and prevent stillbirth contribute to the lack of significant improvement in the stillbirth rate. Currently in 25–40% of stillbirths, the underlying cause cannot be determined and only approximately 20% of stillbirths are potentially predictable in early pregnancy [4–6].

Altered fetal growth and placental abnormalities are the strongest and most prevalent known risk factors for stillbirth [6–8]. However, most pregnancies with placental abnormalities or fetal growth aberrations do not result in stillbirth [7, 8]. An understanding of these interrelationships may contribute to a better understanding of stillbirth mechanisms.

We hypothesized that a combination of certain placental abnormalities and fetal growth aberrations may result in an increased risk of stillbirth. Insight into which placental findings are associated only with stillbirth, only with growth abnormalities, and with both may further our understanding of stillbirth. Fetal growth aberrations and some placental abnormalities are amenable to prenatal detection by ultrasound. Prenatal identification of placental findings and fetal growth abnormalities would likely also improve stillbirth prediction and thus prevention. Delivery effectively prevents stillbirth in pregnancies at high risk of fetal death. In term pregnancies at high risk of stillbirth, delivery is an especially effective intervention for stillbirth prevention, given the minimal risk of neonatal mortality.

## Materials and methods

### Ethics statement

The study was approved by the Institutional Review Boards at each of the clinical recruiting sites and the data coordinating center (Brown University; Emory University; University of Texas Medical Branch at Galveston; University of Texas Health Sciences Center at San Antonio; University of Utah Health Sciences Center; RTI International). All mothers participating in the study gave written informed consent.

### Study design

Women with singleton stillbirths and live births enrolled in the Stillbirth Collaborative Research Network (SCRN) who consented to placental examination were analyzed. The SCRN was a population-based case-control study conducted in five geographic areas in the United States defined by state and county lines: the state of Rhode Island and specific counties in Massachusetts, Georgia, Texas, and Utah. Women were recruited between March 2006 and September 2008 at the time of delivery in 59 community and academic, urban and rural hospitals in the 5 areas with a cumulative average of 80,000 deliveries per year. The hospitals were selected to ensure access to at least 90% of all pregnancies ending in either stillbirth or live birth within each geographic area based on estimates from vital statistics data. All women whose pregnancies resulted in stillbirth and a representative sample of women with live births, oversampled for women delivering live-born infants at <32 weeks’ gestation and those of African-American descent delivering live-born infants at ≥32 weeks’ gestation, were approached for enrollment. Oversampling of live births <32 weeks’ gestation was necessary due to the difference in gestational age distribution between stillbirths and live births, while oversampling of African-American women delivering live births ≥32 weeks’ gestation was undertaken to achieve a ratio of approximately twice the number of live births to stillbirths, similar to the ratio seen among white non-Hispanic women and Hispanic women, out of concern for the increased burden of stillbirth in this population and the potential interaction of race with some hypothesized stillbirth risk factors. Terminations of pregnancy were excluded. Details of the study design have been reported previously [9].

### Stillbirth

Fetal death was defined by Apgar scores of 0 at 1 and 5 minutes, and no signs of life by direct observation, at 20 weeks 0 days of pregnancy or later. This analysis was limited to stillbirths and live births at ≥ 24 weeks 0 days gestation. Births < 24 weeks gestation were excluded to avoid confounding due to variable clinical management of live births at periviable gestational ages. Guidelines for determining grades of maceration were defined in the postmortem examination protocol for intact fetuses as: Grade 0—No maceration, Grade I—Desquamation involving ≤1% of total body surface and brown-red discoloration of umbilical cord stump, Grade II—Desquamation of face, abdomen, or back involving >1% and ≤5% total body surface, Grade III—Desquamation involving >5% of body surface, Grade IV—Total brown skin discoloration, Grade V—Mummification; and for fragmented fetuses as: Grade 0—No maceration, Grade I-II—Tissue appears red/pink and fresh, Grade III—Tissue appears red/pink and mixed with brown, Grade IV—Tissue appears brown/gray, Grade IV—Tissue appears gray.

### Placental abnormalities

The study included a standardized placental examination protocol [10]. This protocol included digital imaging, macroscopic examination, collection of frozen and ambient temperature samples of the cord, membranes and the placental disc, and microscopic examination of sections collected according to a specific sampling protocol. A minimum of five full-thickness placental tissue samples were obtained, one at the umbilical cord insertion and four others determined by random numbers that specified the axes and spacing of sampling. This strategy was used to avoid systematic differences in sampling locations and to ensure dispersion of the tissue sampling sites. All perinatal pathologists participated in training sessions to assure uniform execution of the protocol. The details of placental examination, sample and data collection were described previously [10]. General descriptions of the placental abnormalities investigated are given in Table 1.

**Table 1:** Descriptions of the placental findings.

**General Terms**	**Definitions**
Focal	Present in one area on a single slide.
Multifocal	Present in more than one area and/or in multiple slides.
Patchy	Patches or clusters form when multifocal lesions coalesce to form larger aggregates. In this pattern, the distribution is uneven and all sections do not show the same degree of involvement.
Diffuse	The term diffuse is used when the distribution of lesions involves the full thickness and all the sections to the same degree.
**Placental findings**	**Descriptions**
Single umbilical artery	This is a condition in which a single artery is present in the umbilical cord instead of the usual two arteries.
Velamentous umbilical cord insertion	Normally, the umbilical cord inserts directly into the placenta so that the fetal blood vessels are protected. In velamentous cord insertion, the umbilical cord inserts into the fetal membranes so that the fetal blood vessels travel within the membranes before entering the placental disc. The blood vessels lose the protection provided by Wharton’s substance and are susceptible to injury.
Furcate umbilical cord insertion	In furcate insertion, the umbilical blood vessels lose their protective Wharton’s substance before entering the placental disc. The vessels enter the chorionic plate separately. There are combinations of various types of insertion. It is not infrequent to see a furcate insertion to the placental membranes. This qualifies as both as a furcate and a velamentous insertion.
Circummarginate insertion of the placental membranes	This condition is similar to circumvallate insertion but the marginal white/yellow ring of membrane attachment is flat and does not have a ridge
Circumvallate insertion of the placental membranes	In circumvallate insertion, the membranes are folded over at the margin and decrease the surface area of the placenta. The marginal white/yellow rim of membranes is folded or rolled back to form a distinct raised ridge. This results in doubling of the membranes at the margin and accumulation of fibrin and blood clots.
Terminal (distal) villous immaturity	Terminal villous immaturity is a placental phenotype characterized by enlarged distal (terminal) villi with excessive stroma, hypercellular villous trophoblast, paucity of vasculosyncytial membranes, and a decreased fetoplacental weight ratio.
Terminal (distal) villous hypoplasia	Terminal villous hypoplasia is a decrease in the number and modal diameter of distal villi at the center of the lobule after adjustment for plane of section and gestational age. The decrease in the number of centrilobular distal villi is estimated relative to the number of adjacent stem villi. The extent of terminal villous hypoplasia is estimated as the percentage of villous parenchyma occupied by lobules with centrilobular distal villi that are too few and too small for gestational age in the lower 75% of a full-thickness section.
Acute chorioamnionitis of the placental membranes and chorionic plate	Chorioamnionitis is an inflammation of the fetal membranes due to infection. The presence of inflammatory cells is sufficient to make the diagnosis of chorioamnionitis. No grades or stages were assigned. Presence of polymorphonuclear leukocytes without mononuclear cells indicates acute infection.
Acute funisitis	When the acute inflammatory process involves the Wharton’s substance, a diagnosis of funisitis is made. This is the morphological expression of involvement of the fetal compartment.
Acute umbilical cord arteritis	This is acute infection/inflammation of one or both arteries in the umbilical cord.
Acute umbilical cord phlebitis	This is acute infection/inflammation of the vein in the umbilical cord.
Chorionic plate acute vasculitis of the fetal blood vessels	This is acute infection/inflammation of the fetal blood vessels in the chorionic plate.
Chorionic plate vascular degenerative changes	These changes may be caused by the prolonged presence of meconium within the amniotic cavity. The degeneration consists largely of a homogenization of the muscular wall, which displays eosinophilia, while the nuclei do not stain.
Acute villitis	This is involvement of the chorionic villi with acute inflammatory cells. It ranges from collection of occasional cells to microabscess formation. Pathogenic agents are frequently bacteria such as Listeria monocytogenes.
Chronic villitis	This is involvement of the chorionic villi with chronic inflammatory cells. It can involve plasma cells. It can be specific (i.e. CMV, herpes) or nonspecific (villitis of unknown etiology [VUE]).
Retroplacental hematoma	This is a hematoma behind the placenta. Abruptio placenta is defined as detachment of the placenta from the inner wall of the uterus. Pathologically this results in a retroplacental hematoma. Eventually the villous tissue underlying the hematoma infarct. If they are fresh (less than two hours) it may be difficult to distinguish them from normal postpartum blood clots. After several hours however, the retroplacental clot will become adherent to the maternal surface and identifiable on macroscopic examination. Compression of the underlying villous tissue then follows in a few hours.
Parenchymal infarction	Placental infarcts are the most common lesions seen in the placenta. They represent dead villous tissue due to compromised intervillous (maternal) circulation. When parenchymal infarcts are identified away from the placental margins and particularly when they are randomly distributed, placental compromised perfusion is confirmed. Parenchymal infarcts in any location in the first and second trimester placentas are always abnormal.
Intraparenchymal (intervillous) thrombus	Intraparenchymal thrombi are common parenchymal lesions. They are localized clots in the intervillous space. They frequently develop in the maternal circulation due to increased thrombosis in the setting of maternal thrombophilias and preeclampsia. Some intraparenchymal thrombi may occur secondary to leakage from the fetal capillaries resulting in fetal maternal hemorrhage.
Perivillous, intervillous fibrin, fibrinoid deposition patterns	This is perivillous fibrin encasing of chorionic villi. It often accompanies a significant increase of fibrin deposition in the basal plate and can present with or without extravillous trophoblastic proliferation of the villi. Maternal floor infarction/ Gitterinfarkt is a specific pattern of massive perivillous fibrin deposition with extensive involvement of terminal villi in the basal portion of the placenta by a surrounding matrix of varying proportions of pink fibrinoid material containing extravillous trophoblast and true fibrin often arranged in swirling whorls and fascicles.
Fetal vascular thrombi in the chorionic plate	These are thrombi identified in the chorionic plate fetal blood vessels. Whatever the etiology, the presence of even one thrombus is considered significant. If this lesion is associated with downstream avascular villi, it is usually not related to infection and called fetal thrombotic vasculopathy. (After fetal death, fetal circulation stops and fetal vascular thrombi are not formed).
Avascular villi	Loss of villous capillaries and bland hyalinization of the villous stroma in a distribution consistent with a single upstream occlusive event. This is different than the villous changes seen with fetal death.
Placental edema	The villous tissue is diffusely boggy, and friable. There are pools of extracellular fluid expanding the stromal area and often accompanied by increased Hofbauer cells, cytotrophoblast hyperplasia, and artifactual dehiscence of trophoblast from stroma. When seen with hydrops fetalis, it is considered a lethal lesion.

### Fetal growth abnormalities

Gestational age (GA) at death (for stillbirths) or delivery (for live births) was estimated using an algorithm developed by the SCRN investigators [11]. Preterm birth was defined as GA at delivery or death < 37 weeks 0 days. Birth weight percentiles were determined using birth weight, the SCRN estimate of GA at death (stillbirths) or delivery (live births), and an expected weight for GA based on ultrasound norms [12]. Ultrasound norms were used for both live births and stillbirths because they were derived using ultrasound measurements from ongoing uncomplicated pregnancies and have been shown to identify more babies as abnormally grown compared to population norms which are derived from birth weights of all live births, including preterm and/or with growth abnormalities, and underestimate the proportion of babies with abnormal growth [8, 13].

Small for gestational age (SGA) was defined as birth weight less than the 10^th^ percentile, average for gestational age (AGA) as birth weight between the 10^th^ and 90^th^ percentiles, and large for gestational age (LGA) as birth weight above the 90^th^ percentile.

### Statistical analysis

Analysis was restricted to singletons with an adequate placental examination. Fragmented placentas and placentas from mummified stillbirths were excluded. Mummified stillbirths were defined as fragmented babies who had Grade IV-V maceration and intact babies who had Grade V maceration. In addition, stillbirths and live births with missing birth weight or with GA < 24 weeks by SCRN algorithm were excluded.

Data were weighted to take into account the study design and other aspects of the sampling plus differential availability of an adequate placental examination. Weighted analyses were conducted using SUDAAN version 11.0.1 software. Proportions of stillbirths and live births with selected maternal and infant characteristics and with each placental finding were compared with statistical significance determined by the Wald chi-square test. Comparisons of the continuous measures placental weight and ratio birth weight to placental weight were based on weighted ranks and conducted using regression models that included the stillbirth indicator (stillbirth/live birth) only. Next, tests of association between each placental finding and birth weight percentile were conducted for stillbirths and live births separately with statistical significance determined by the adjusted Wald F test. Regression models used for comparisons of continuous measures based on weighted ranks included the percentile group indicator only.

Subsequently, tests for whether the association between each placental finding and birth weight percentile differed for stillbirths and live births were conducted using a logistic or linear regression model with a placental finding as the dependent variable and the percentile group indicator, stillbirth indicator, and effects for the interaction as independent variables, with the p-value associated with the interaction between percentile group and stillbirth indicator reported. Where a placental finding was not noted for any stillbirths or live births in a birth weight percentile category (or conversely when noted for all in a category), the observation in that percentile group with the smallest analysis weight was recoded as having the finding (or not having the finding) for the purpose of providing an upper bound on the p-value for the interaction in the presence of these zero cells. An asterisk next to the p-value indicates it was derived in this manner. All proportions reported, as well as all other p-values, were based on the data without recoding zero cells. In each case where the interaction p-value was approximated by recoding zero cells, the revised proportions were checked to verify that the recoding did not introduce more extreme results than were present in the original data. Examples are provided in S1 Table.

In the primary analyses, comparisons of placental weight and the ratio birth weight/placental weight were additionally conducted with adjustment for GA. Linear regression models that included GA as a continuous variable and models that included GA as a categorical variable (groups as noted in table footnotes) were compared with similar inference. P-values from models that included categorical GA are reported in brackets beside unadjusted p-values in the tables.

## Results

Consent for placental exam was obtained for 613 of the 620 singleton stillbirth pregnancies and for 1,747 of the 1,871 singleton live birth pregnancies. Ninety-five stillbirth placentas and 547 live birth placentas were excluded from analysis because of inadequate examination, fragmentation, or mummification, leaving 518 stillbirth placentas and 1,200 live birth placentas. From these, 199 stillbirths (16 missing birth weight, 183 with GA <24 weeks) and 81 live births (17 missing birth weight, 64 with GA <24 weeks) were excluded. Thus, 319 singleton stillbirths and 1119 singleton live births with a GA of ≥ 24 weeks at death or delivery were studied.

A greater proportion of women who delivered stillbirths had hypertension before pregnancy (11% versus 5%, p<0.001), gestational hypertension/pre-eclampsia (20% versus 11%, p<0.001), and pregestational diabetes (7% versus 2%, p<0.001) (Table 2). Stillborn infants had lower GA compared to live born infants (median 33 versus 39 weeks, p<0.001) and lower birth weight (median 1,949 versus 3,321 grams, p<0.001) and a larger proportion had a congenital anomaly (13% versus 3%, p<0.001) (Table 2). Less than a third of the 319 stillborn infants were born at term (unweighted n = 88; weighted n = 87, 28%) while the majority of the 1119 live births were born at term (unweighted n = 942; weighted n = 854, 90%). A median of 2 days elapsed between estimated death and delivery of stillborn infants (interquartile range 1 to 4 days). No maceration was reported for 36 (13%) of the 282 stillbirths with information, Grade I maceration for 41 (15%), Grade II for 65 (23%), Grade III for 120 (43%), and Grade IV for 20 (7%). No consent for postmortem exam was given for 36 stillborn infants and information about maceration information was missing for one.

**Table 2:** Characteristics of singleton stillbirths and live births ≥ 24 weeks GA with adequate placental examination.

Characteristic, column % or as shown ^1/^	Stillbirth	Live birth	P-value ^2/^
Unweighted number	319	1119	
Weighted number	314	948	
MOTHER			
Age at delivery, years			
Median	26	27	0.59
Interquartile range	22 to 32	22 to 31	
Race/ethnicity			0.02
Non-Hispanic white	36	45	
Non-Hispanic black	17	11	
Hispanic	41	36	
Other	6	7	
Chronic hypertension	11	5	0.007
Gestational hypertension/pre-eclampsia	20	11	<0.001
Pre-gestational diabetes	7	2	<0.001
Gestational diabetes	7	8	0.63
INFANT			
GA, weeks			<0.001
Median	33	39	
Interquartile range	28 to 37	38 to 40	
GA category			<0.001
24–31 wk	41	1	
32–26 wk	31	8	
37+ wk	28	90	
Birth weight, grams			<0.001
Median	1,949	3,321	
Interquartile range	984 to 2,873	3,005 to 3,638	
Male sex	55	50	0.13
Congenital anomaly	13	3	<0.001

^1/^ Weighted percentages and other statistics are shown. Information was missing as follows (unweighted n): pre-pregnancy hypertension, 4; pre-pregnancy diabetes, 4; male sex, 2.

^2/^ P-value for a difference between stillbirths and live births by the Wald chi-square test (categorical variables) or the median test (continuous variables).

**Fig 1:**
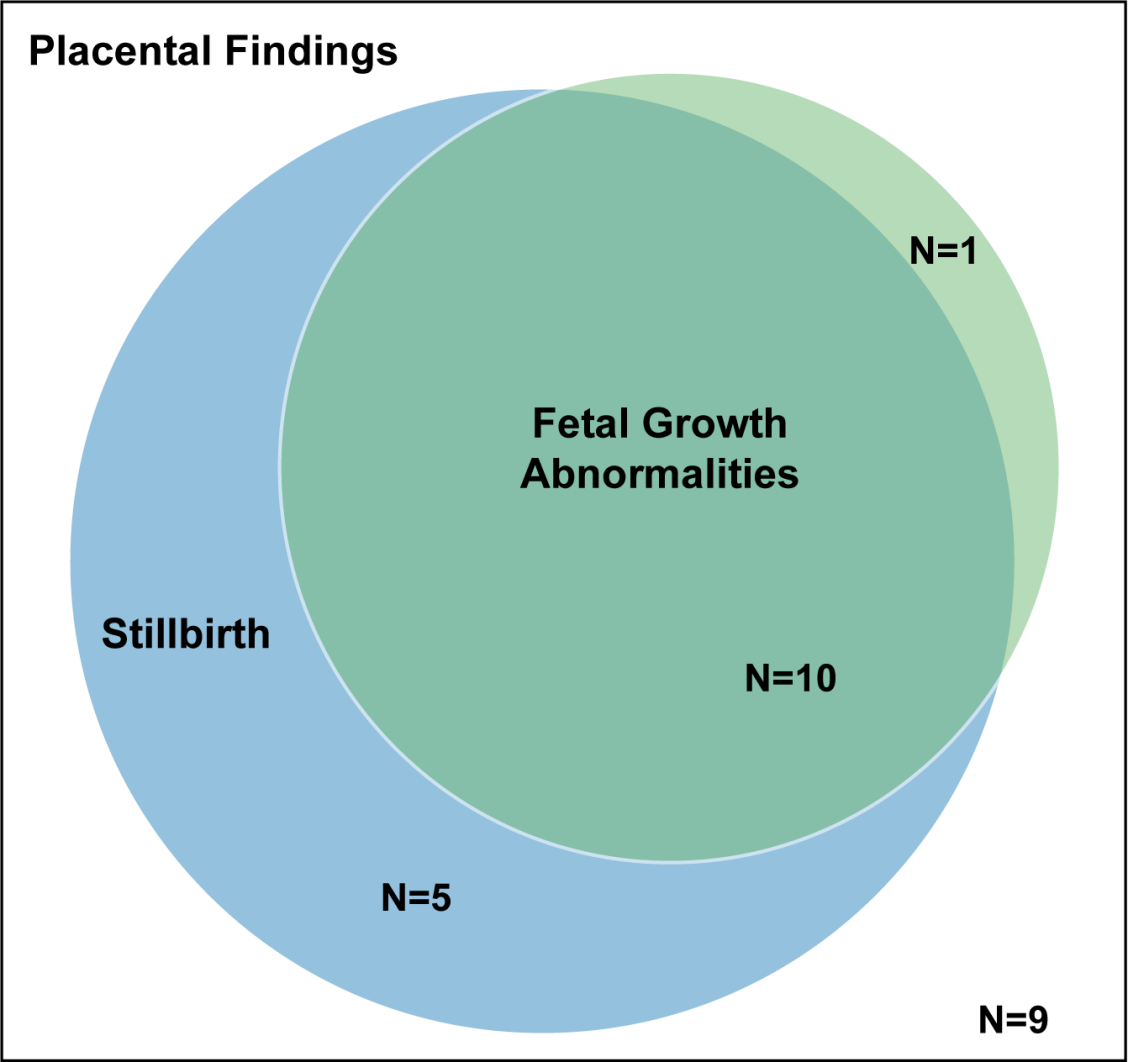
The relationship between 25 placental abnormalities studied, fetal growth, and stillbirth. A total of 15 placental findings were associated with an increased risk of stillbirth of which 10 were also associated with fetal growth abnormalities. Terminal villous hypoplasia (TVH) was associated with fetal growth but not stillbirth.

The placental examination investigated 25 findings, of which 15 were significantly associated with stillbirth (Table 3). The 13 significant findings reported as percentages were more common among stillbirths than live births. Median placental weight and median birth weight to placental weight ratio were each significantly lower in stillbirths than in live births. Ten of the 15 placental findings associated with stillbirth were also associated with fetal growth abnormalities (Fig 1; single umbilical artery, velamentous insertion, terminal villous immaturity, retroplacental hematoma, parenchymal infarction, intraparenchymal thrombus, avascular villi, placental edema, placental weight, and ratio birth weight/placental weight) while 5 of the 15 findings more common in stillbirths were not associated with fetal growth abnormalities (acute chorioamnionitis of placental membranes; acute chorioamionitis of chorionic plate; chorionic plate vascular degenerative changes; perivillous, intervillous fibrin, fibrinoid deposition; fetal vascular thrombi in the chorionic plate). Terminal villous hypoplasia was the only placental finding associated with fetal growth that was not significantly more common among stillbirths than among live births (Fig 1; Table 3).

**Table 3:** Placental findings in singleton stillbirths and live births ≥ 24 weeks GA.

Characteristic, column % or as shown ^1/^	Stillbirth	Live birth	P-value ^2/^
Unweighted number	319	1119	
Weighted number	314	948	
DEVELOPMENTAL DISORDERS			
Umbilical cord			
Single umbilical artery	8.4	1.7	<0.001
Velamentous insertion	3.9	1.2	0.02
Furcate insertion	1.9	3.5	0.12
Placental membranes			
Circummarginate insertion	13.4	10.8	0.23
Circumvallate insertion	2.2	1.4	0.40
Fetal villous capillaries			
Terminal villous immaturity (diffuse)	10.1	2.3	<0.001
Terminal villous hypoplasia (diffuse)	3.4	1.8	0.21
INFLAMMATORY DISORDERS			
Maternal inflammatory response			
Acute chorioamnionitis—placental membranes	24.5	11.9	<0.001
Acute chorioamnionitis—chorionic plate	17.3	11.8	0.02
Fetal inflammatory response			
Acute funisitis	3.0	3.2	0.86
Acute umbilical cord arteritis (one or more arteries)	0.6	1.7	0.05
Acute umbilical cord phlebitis	2.5	2.9	0.65
Chorionic plate acute vasculitis	3.0	5.0	0.10
Chorionic plate vascular degenerative changes	5.7	0.5	<0.001
Villitis			
Acute diffuse villitis	0.8	0.1	0.16
Chronic diffuse villitis	2.1	0.5	0.07
CIRCULATORY DISORDERS			
Maternal circulatory disorders			
Retroplacental hematoma	17.1	4.2	<0.001
Parenchymal infarction	33.3	15.8	<0.001
Intraparenchymal thrombus	22.7	13.5	<0.001
Perivillous, intervillous fibrin, fibrinoid deposition (diffuse)	9.2	1.5	<0.001
Fetal circulatory disorders			
Fetal vascular thrombi in the chorionic plate	28.6	7.1	<0.001
Avascular villi	19.4	6.9	<0.001
Placental edema	4.0	1.0	0.01
Placental weight, median (IQR)	300 (185–414)	435 (376–508)	<0.001
Ratio birth weight/placental weight, median (IQR)	6.3 (4.9–8.0)	7.5 (6.7–8.5)	<0.001

^1/^ Weighted percentages and other statistics are shown. Information was missing as follows (unweighted n): single umbilical artery, 8; velamentous insertion, 27; furcate insertion, 33; circummarginate insertion, 46; circumvallate insertion, 46; terminal villous immaturity, 9; terminal villous hypoplasia, 13; any developmental disorder, 74; acute chorioamnionitis—placental membraines, 18; acute chorioamnionitis—chorionic plate, 15; acute funisitis, 11; acute umbilical cord arteritis, 31; acute umbilical cord phlebitis, 16; chorionic plate acute vasculitis, 16; chorionic plate vascular degenerative changes, 17; acute diffuse villitis, 7; chronic diffuse villitis, 8; any inflammatory disorder, 46; retroplacental hematoma, 8; parenchymal infarction, 7; intraparenchymal thrombus, 12; perivillous, intervillous fibrin, fibrinoid deposition, 64; any maternal circulatory disorder, 44; fetal vascular thrombi in the chorionic plate, 10; avascular villi, 7; edema, 10; any fetal circulatory disorder, 13; placental weight, 14; ration birth weight/placental weight, 14.

^2/^ P-value for an association between the placental finding and stillbirth by the Wald chi-square test based on observed minus expected frequencies for categorical variables. For each continuous measurement, the test is for mean difference in weighted ranks.

The associations between placental findings, fetal growth abnormalities, and stillbirth showed five distinct patterns: placental findings associated with (1) stillbirth but not fetal growth abnormalities; (2) fetal growth abnormalities in stillbirths only; (3) fetal growth abnormalities in live births only; (4) fetal growth abnormalities in stillbirths and live births in a similar manner; (5) a different pattern of fetal growth abnormalities in stillbirths and live births (Table 4).

**Table 4:** Patterns of association between placental findings, fetal growth and stillbirth.

PLACENTAL FINDINGS
***(1)* associated with stillbirth but not fetal growth**
Acute chorioamnionitis of placental membranes
Acute chorioamnionitis of chorionic plate
Chorionic plate vascular degenerative changes
Perivillous, intervillous fibrin, fibrinoid deposition
Fetal vascular thrombi in the chorionic plate
***(2)* associated with fetal growth abnormalities in stillbirths only**
Velamentous umbilical cord insertion in preterm pregnancies
Terminal villous hypoplasia in preterm pregnancies
Parenchymal infarction in preterm pregnancies
***(3)* associated with fetal growth abnormalities in live births only**
Single umbilical artery in term pregnancies
Terminal villous hypoplasia in term pregnancies*
Parenchymal infarction in term pregnancies
***(4)* associated with fetal growth abnormalities in stillbirths and live births in a similar manner**
Placental weight in preterm pregnancies
Intraparenchymal thrombus in term pregnancies
***(5)* with a different pattern of fetal growth abnormalities in stillbirths and live births**
Terminal villous immaturity (diffuse) in preterm pregnancies
Retroplacental hematoma in preterm and term pregnancies
Avascular villi in preterm pregnancies
Placental edema in preterm pregnancies
Placental weight in term pregnancies
Ratio birth weight/placental weight in term pregnancies

* Terminal villous hypoplasia is almost twice as common in stillbirths (3.4%) as in live births (1.8%), but this difference did not reach statistical significance.

(1) Placental findings associated with stillbirth but not fetal growth abnormalities.

Acute chorioamnionitis of the placental membranes and chorionic plate; chorionic plate vascular degenerative changes; perivillous or intervillous fibrin, fibrinoid depositions; and fetal vascular thrombi in the chorionic plate were more common among stillbirths than among live births (Table 3). Although associated with stillbirth, no significant associations were found between these placental findings and fetal growth abnormalities in either preterm stillbirths and live births (Table 5) or term stillbirths and live births (Table 6).

**Table 5:** Placental findings in preterm (24–36 weeks) stillbirths and live births small, average, and large for gestational age.^1/^

Characteristic, column % or as shown ^2/^	SGA	AGA	LGA	P-value for association, SBs & LBs separately^3/^	P-value for interaction ^4/^
Unweighted number of stillbirths	122	87	22		
Weighted number of stillbirths	121	84	22		
Unweighted number of live births	46	113	18		
Weighted number of live births	23	58	13		
DEVELOPMENTAL DISORDERS					
Umbilical cord					
Single umbilical artery					0.24*
Stillbirths	8.4	10.7	10.8	0.85	
Live births	3.0	1.1	0.0	0.41	
Velamentous insertion					0.47*
Stillbirths	5.2	4.7	0.0	0.03	
Live births	2.9	2.4	0.0	0.21	
Furcate insertion					0.07*
Stillbirths	1.7	1.5	0.0	0.26	
Live births	0.0	2.2	8.7	0.27	
Placental membranes					
Circummarginate insertion					0.99
Stillbirths	10.6	14.9	22.0	0.38	
Live births	4.8	7.3	12.1	0.73	
Circumvallate insertion					0.21*
Stillbirths	2.3	3.0	3.8	0.91	
Live births	0.0	1.6	0.0	0.32	
Fetal villous capillaries					
Terminal villous immaturity (diffuse)					0.009*
Stillbirths	7.3	8.1	19.6	0.44	
Live births	7.2	1.7	0.0	0.22	
Terminal villous hypoplasia (diffuse)					0.17*
Stillbirths	7.1	0.0	0.0	0.03	
Live births	10.8	1.7	0.0	0.21	
INFLAMMATORY DISORDERS					
Maternal inflammatory response					
Acute chorioamnionitis—placental membranes					0.87
Stillbirths	28.7	19.4	16.7	0.22	
Live births	26.5	12.5	14.3	0.71	
Acute chorioamnionitis—chorionic plate					0.95
Stillbirths	16.3	13.1	20.1	0.68	
Live births	21.8	15.0	28.1	0.63	
Fetal inflammatory response					
Acute funisitis					0.55*
Stillbirths	2.7	4.1	0.0	0.06	
Live births	2.6	4.0	0.6	0.13	
Acute umbilical cord arteritis					0.69*
Stillbirths	0.0	0.0	4.6	0.60	
Live births	1.5	3.0	3.9	0.60	
Acute umbilical cord phlebitis					0.83
Stillbirths	0.7	2.8	4.6	0.42	
Live births	2.1	3.9	10.5	0.43	
Chorionic plate acute vasculitis					0.22
Stillbirths	1.6	2.8	3.4	0.80	
Live births	1.9	8.4	0.6	0.10	
Chorionic plate vascular degenerative changes					0.99*
Stillbirths	3.4	7.5	11.1	0.26	
Live births	0.0	0.2	0.0	0.61	
Villitis					
Acute diffuse villitis					0.88*
Stillbirths	1.5	0.9	0.0	0.27	
Live births	0.0	0.1	0.0	0.61	
Chronic diffuse villitis					0.44*
Stillbirths	3.4	0.0	0.0	0.14	
Live births	2.5	0.3	0.0	0.23	
CIRCULATORY DISORDERS					
Maternal circulatory disorders					
Retroplacental hematoma					0.03*
Stillbirths	26.7	16.9	0.0	<0.001	
Live births	5.7	2.7	15.3	0.39	
Parenchymal infarction					0.40
Stillbirths	48.1	25.9	14.6	<0.001	
Live births	17.6	16.1	7.2	0.52	
Intraparenchymal thrombus					0.34
Stillbirths	17.9	23.2	25.4	0.58	
Live births	14.4	11.1	34.6	0.32	
Perivillous, intervillous fibrin, fibrinoid deposition					0.43
Stillbirths	14.8	8.1	5.8	0.25	
Live births	6.4	1.7	0.3	0.34	
Fetal circulatory disorders					
Fetal vascular thrombi in the chorionic plate					0.91
Stillbirths	21.8	26.9	33.3	0.51	
Live births	8.8	9.4	8.8	1.00	
Avascular villi					0.04*
Stillbirths	19.3	21.3	16.8	0.87	
Live births	4.6	8.4	0.0	0.09	
Placental edema					0.01*
Stillbirths	3.2	2.3	11.1	0.51	
Live births	0.5	3.8	0.0	0.15	
Placental weight					0.66 [0.90]
Stillbirths				<0.001 [<0.001]	
Median (IQR)	180 (134–247)	281 (208–390)	421 (306–513)		
Live births				<0.001 [<0.001]	
Median (IQR)	318 (234–330)	361 (301–425)	483 (396–573)		
Ratio birth weight/placental weight					0.32 [0.40]
Stillbirths				0.62 [0.45]	
Median (IQR)	5.5 (4.1–7.7)	6.1 (4.6–7.0)	6.0 (4.7–7.6)		
Live births				0.17 [0.44]	
Median (IQR)	6.1 (5.9–6.9)	6.8 (5.7–7.8)	6.5 (5.9–7.4)		

^1/^ Birth weight percentiles for GA were determined using Hadlock ultrasound norms and GA at death (stillbirths) or delivery (live births) by the SCRN algorithm.

^2/^ Weighted percentages and other statistics are shown. For stillbirths, information was missing as follows (unweighted n): single umbilical artery, 1; velamentous insertion, 2; furcate insertion, 3; circummarginate insertion, 8; circumvallate insertion, 8; terminal villous immaturity, 3; terminal villous hypoplasia, 3; any developmental disorder, 12; acute chorioamnionitis—chorionic plate, 2; acute funisitis, 1; acute umbilical cord arteritis, 5; acute umbilical cord phlebitis, 3; chorionic plate acute vasculitis, 7; chorionic plate vascular degenerative changes, 7; acute diffuse villitis, 1; chronic diffuse villitis, 2; any inflammatory disorder, 10; retroplacental hematoma, 4; parenchymal infarction, 3; intraparenchymal thrombus, 2; perivillous, intervillous fibrin, fibrinoid deposition, 12; any maternal circulatory disorder, 8; fetal vascular thrombi in the chorionic place, 5; avascular villi, 2; edema, 6; any fetal circulatory disorder, 6; placental weight, 3; ratio birth weight/placental weight, 3. For live births, information was missing as follows (unweighted n): velamentous insertion, 4; furcate insertion, 5; circummarginate insertion, 8; circumvallate insertion, 8; terminal villous immaturity, 1; terminal villous hypoplasia, 3; any developmental disorder, 13; acute chorioamnionitis—placental membranes, 2; acute chorioamnionitis—chorionic plate, 2; acute umbilical cord arteritis, 4; chorionic plate acute vasculitis, 1; chorionic plate vascular degenerative changes, 1; acute diffuse villitis, 2; chronic diffuse villitis, 2; any inflammatory disorder, 1; retroplacental hematoma, 1; parenchymal infarction, 1; intraparenchymal thrombus, 1; perivillous, intervillous fibrin, fibrinoid deposition, 14; any maternal circulatory disorder, 10; fetal vascular thrombi in the chorionic plate, 1; avascular villi, 1; any fetal circulatory disorder, 1.

^3/^ P-value by the adjusted Wald F test for an association between a placental finding and birth weight percentile among stillbirths and live births separately. For each continuous measurement, the test is for mean difference in weighted ranks; p-values after adjustment for GA (24–31, 32–36) are shown in brackets.

^4/^ P-value by the adjusted Wald F test for whether the association between a placental finding and birth weight percentile differs for stillbirths and live births. For each continuous measure, the test was based on weighted ranks; p-values after adjustment for GA (24–31, 32–36) are shown in brackets.

* None of the births in one or more group had the placental finding. The p-value reported was estimated as described in Statistical Analysis.

**Table 6:** Placental findings in term (37+ weeks) stillbirths and live births small, average, and large for gestational age.^1/^

Characteristic, column % or as shown ^2/^	SGA	AGA	LGA	P-value for association, SBs & LBs separately ^3/^	P-value for interaction^4/^
Unweighted number of stillbirths	28	50	10		
Weighted number of stillbirths	28	50	9		
Unweighted number of live births	156	729	57		
Weighted number of live births	138	666	50		
DEVELOPMENTAL DISORDERS					
Umbilical cord					
Single umbilical artery					0.69*
Stillbirths	3.7	7.4	0.0	0.16	
Live births	1.9	1.9	0.0	0.002	
Velamentous insertion					0.93*
Stillbirths	3.8	1.9	0.0	0.42	
Live births	1.0	0.9	2.8	0.65	
Furcate insertion					0.46*
Stillbirths	8.9	0.0	0.0	0.36	
Live births	5.4	3.3	2.6	0.57	
Placental membranes					
Circummarginate insertion					0.90
Stillbirths	13.3	12.7	18.6	0.90	
Live births	8.9	11.5	13.8	0.61	
Circumvallate insertion					0.61*
Stillbirths	0.0	1.4	0.0	0.61	
Live births	1.6	1.4	1.2	0.97	
Fetal villous capillaries					
Terminal villous immaturity (diffuse)					0.70*
Stillbirths	14.8	14.9	0.0	0.05	
Live births	1.1	2.5	1.2	0.30	
Terminal villous hypoplasia (diffuse)					0.45*
Stillbirths	3.5	1.9	0.0	0.42	
Live births	3.4	1.4	0.0	0.006	
INFLAMMATORY DISORDERS					
Maternal inflammatory response					
Acute chorioamnionitis—placental membranes					0.63
Stillbirths	19.4	27.1	36.9	0.54	
Live births	12.0	10.5	21.7	0.26	
Acute chorioamnionitis—chorionic plate					0.09
Stillbirths	11.9	28.6	16.4	0.17	
Live births	12.6	10.2	18.1	0.36	
Fetal inflammatory response					
Acute funisitis					0.87*
Stillbirths	2.7	4.1	0.0	0.30	
Live births	2.8	2.8	9.7	0.43	
Acute umbilical cord arteritis (one or more arteries)					0.69*
Stillbirths	2.7	0.0	0.0	0.60	
Live births	1.1	1.6	2.0	0.83	
Acute umbilical cord phlebitis					0.45*
Stillbirths	2.7	5.7	0.0	0.22	
Live births	3.5	2.1	9.5	0.17	
Chorionic plate acute vasculitis					0.99*
Stillbirths	6.1	5.4	0.0	0.16	
Live births	5.5	4.6	7.3	0.71	
Chorionic plate vascular degenerative changes					0.95*
Stillbirths	6.5	6.2	0.0	0.17	
Live births	0.8	0.5	0.0	0.17	
Villitis					
Acute diffuse villitis					1.00*
Stillbirths	0.0	0.0	0.0		
Live births	0.0	0.2	0.0	0.61	
Chronic diffuse villitis					0.78*
Stillbirths	3.8	2.5	0.0	0.41	
Live births	0.0	0.6	0.0	0.14	
CIRCULATORY DISORDERS					
Maternal circulatory disorders					
Retroplacental hematoma					0.02*
Stillbirths	23.0	1.9	0.0	0.05	
Live births	2.0	4.7	2.0	0.13	
Parenchymal infarction					0.26
Stillbirths	35.2	18.7	20.0	0.31	
Live births	17.5	16.3	5.4	0.005	
Intraparenchymal thrombus					0.72
Stillbirths	10.3	34.4	49.7	0.01	
Live births	5.1	14.9	16.7	<0.001	
Perivillous, intervillous fibrin, fibrinoid deposition					0.62*
Stillbirths	9.4	0.0	0.0	0.37	
Live births	2.6	1.1	1.1	0.57	
Fetal circulatory disorders					
Fetal vascular thrombi in the chorionic plate					0.66
Stillbirths	45.8	30.5	56.3	0.23	
Live births	9.5	6.2	7.6	0.49	
Avascular villi					0.18
Stillbirths	32.8	10.2	18.8	0.09	
Live births	8.1	6.7	6.5	0.84	
Placental edema					0.81*
Stillbirths	0.0	7.0	9.9	0.14	
Live births	0.7	0.9	1.2	0.94	
Placental weight					0.03 [0.03]
Stillbirths, median (IQR)	385 (329–439)	434 (353–484)	466 (381–648)	0.01 [0.003]	
Live births, median (IQR)	364 (313–406)	451 (400–516)	554 (502–610)	<0.001 [<0.001]	
Ratio birth weight/placental weight					0.02 [0.02]
Stillbirths, median (IQR)	6.8 (6.1–7.8)	7.7 (7.0–8.7)	9.3 (6.0–10.5)	0.04 [0.05]	
Live births, median (IQR)	7.7 (6.8–8.8)	7.6 (6.8–8.5)	7.4 (6.8–8.5)	0.53 [0.52]	

^1/^ Birth weight percentiles for GA were determined using Hadlock ultrasound norms and GA at death (stillbirths) or delivery (live births) by the SCRN algorithm.

^2/^ Weighted percentages and other statistics are shown. For stillbirths, information was missing as follows (unweighted n): velamentous insertion, 1; furcate insertion, 2; circummarginate insertion, 3; circumvallate insertion, 3; terminal villous immaturity, 1; terminal villous hypoplasia, 2; any developmental disorder, 4; acute chorioamnionitis—placental membranes, 1; acute umbilical cord arteritis, 1; chorionic plate acute vasculitis, 2; chorionic plate vascular degenerative changes, 2; any inflammatory disorder, 3; intraparenchymal thrombus, 1; perivillous, intervillous fibrin, fibrinoid deposition, 9; any maternal circulatory disorder, 1; edema, 1; any fetal circulatory disorder, 1. For live births, information was missing as follows (unweighted n): single umbilical artery, 7; velamentous insertion, 20; furcate insertion, 23; circummarginate insertion, 27; circumvallate insertion, 27; terminal villous immaturity, 4; terminal villous hypoplasia, 5; any developmental disorder, 45; acute chorioamnionitis—placental membranes, 15; acute chorioamnionitis—chorionic plate, 11; acute funisitis, 10; acute umbilical cord arteritis, 21; acute umbilical cord phlebitis, 13; chorionic plate acute vasculitis, 6; chorionic plate vascular degenerative changes, 7; acute diffuse villitis, 4; chronic diffuse villitis, 4; any inflammatory disorder, 32; retroplacental hematoma, 3; parenchymal infarction, 3; intraparenchymal thrombus, 8; perivillous, intervillous fibrin, fibrinoid deposition, 29; any maternal circulatory disorder, 25; fetal vascular thrombi in the chorionic plate, 4; avascular villi, 4; edema, 3; any fetal circulatory disorder, 5; placental weight, 11; ratio birth weight/placental weight, 11.

^3/^ P-value by the adjusted Wald F test for an association between a placental finding and birth weight percentile among stillbirths and live births separately. For each continuous measurement, the test is for mean difference in weighted ranks; p-values after adjustment for GA (37–38, 39+) are shown in brackets.

^4/^ P-value by the adjusted Wald F test for whether the association between a placental finding and birth weight percentile differs for stillbirths and live births. For each continuous measure, the test was based on weighted ranks; p-values after adjustment for GA (37–38, 39+) are shown in brackets.

* None of the births in one or more group had the placental finding. The p-value reported was estimated as described in Statistical Analysis.

(2) Placental findings associated with fetal growth abnormalities in stillbirths only.

Velamentous umbilical cord insertion, terminal villous hypoplasia, and parenchymal infarction were significantly associated with fetal growth abnormalities in preterm stillbirths but a significant association was not found in preterm live births (Table 5). The proportion with each of these findings was larger in SGA preterm stillbirths than in AGA and LGA preterm stillbirths. In term stillbirths the proportion with each of these findings was also highest in those who were SGA. However, significant associations with fetal growth were not observed in term stillbirths (Table 6).

(3) Placental findings associated with fetal growth abnormalities in live births only.

Single umbilical artery, terminal villous hypoplasia and parenchymal infarction were significantly associated with fetal growth abnormalities in term live births but not in term stillbirths (Table 6). Each of these findings was either not observed or less common in LGA than in AGA and SGA term live births. Although each of these findings was similarly not observed or less common in preterm LGA compared to AGA and SGA live births, statistically significant differences were not observed in preterm live births (Table 5).

(4) Placental findings associated with fetal growth abnormalities in stillbirths and live births in a similar manner.

Placental weight in preterm pregnancies and intraparenchymal thrombus in term pregnancies were significantly associated with fetal growth abnormalities in both stillbirths and live births in a similar manner (Tables 5 and 6). These two placental findings were respectively higher or more common in LGA than in AGA and SGA stillbirths and live births.

(5) Placental findings with a different pattern of fetal growth abnormalities in stillbirths and live births.

Among preterm pregnancies, patterns of association between fetal growth abnormalities and each of terminal villous immaturity, avascular villi, placental edema and retroplacental hematoma differed for stillbirths and live births (Table 5, significant p value for interaction). Terminal villous immaturity, avascular villi, and placental edema were not observed among preterm LGA live births but were common among preterm LGA stillbirths. Retroplacental hematoma was significantly associated with fetal growth among preterm stillbirths with a quarter of SGA and none of the LGA preterm stillbirths demonstrating retroplacental hematoma. Among preterm live births, the proportion with retroplacental hematoma was highest among those with LGA pregnancies (Table 5).

**Fig 2:**
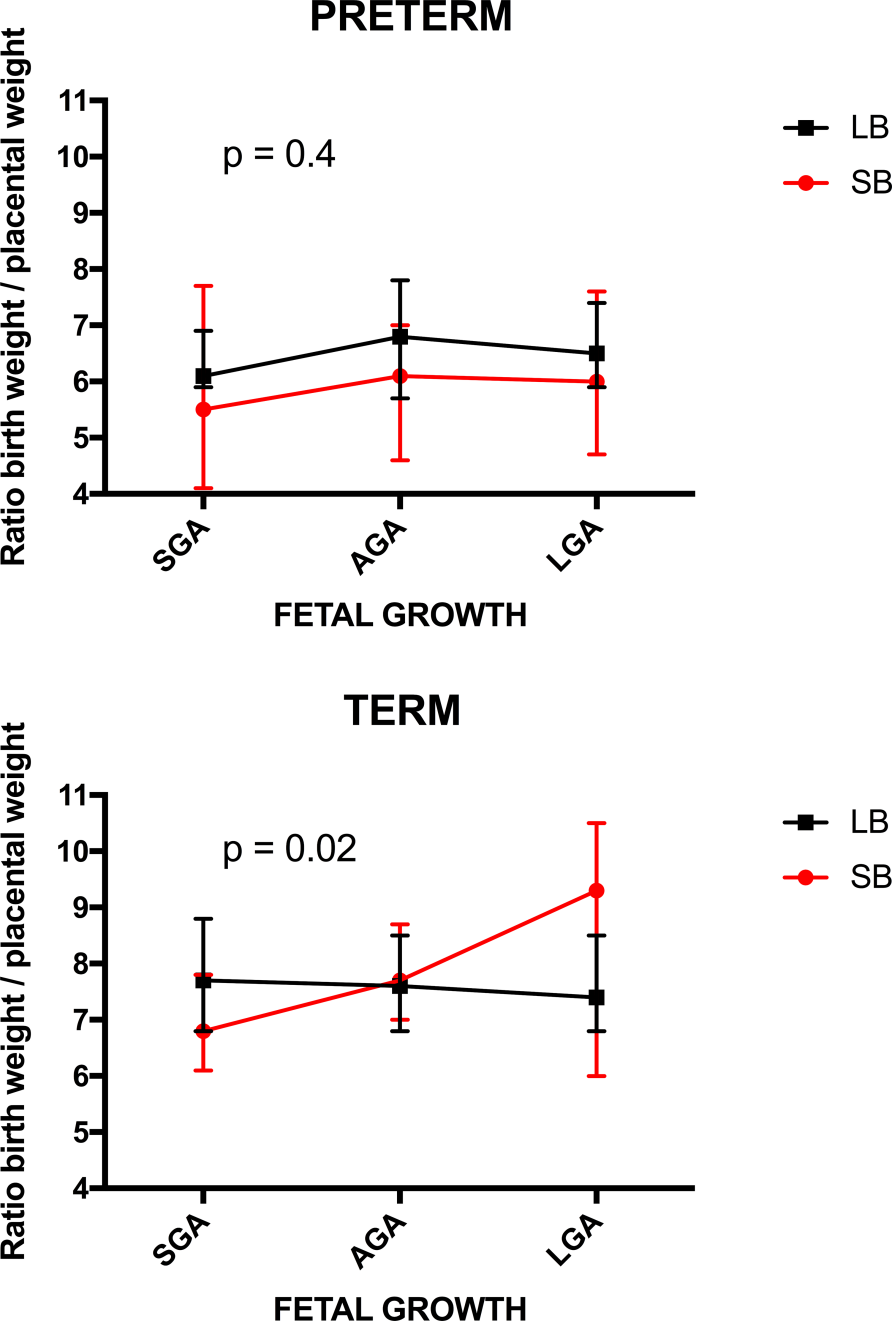
Birth weight to placental weight ratio in preterm and term pregnancies. P-value for a test of whether the association between the ratio and birth weight percentile group differed for stillbirths and live births after adjustment for gestational age is shown.

In term pregnancies, different fetal growth patterns were observed in stillbirths and live births for retroplacental hematoma, placental weight, and the ratio of birth weight to placental weight (Table 6, significant p value for interaction). Retroplacental hematoma was found most frequently in term SGA stillbirths and was not observed among term LGA stillbirths but was observed among term LGA live births. In term stillbirths, the median placental weight was 20g larger in SGA, 40g smaller in AGA and 90g smaller in LGA than in term live births (Table 6). Median birth weight to placental weight ratio was almost identical for all fetal growth categories among term live births. The ratio was markedly smaller among term SGA stillbirths (median (IQR): 6.8 (6.1–7.8) vs. 7.7 (6.8–8.8), respectively) and substantially larger in term LGA stillbirths compared to term live births (median (IQR): 9.3 (6.0–10.5) vs. 7.4 (6.8–8.5), respectively) (Table 6; Fig 2). Among preterm pregnancies, there was no significant association between birth weight to placental weight ratio and the birth weight categories among stillbirths or live births nor was there a difference in this association between stillbirths and live births. (Table 5; Fig 2).

Associations between placental findings and fetal growth among preterm births were essentially similar in the subsets of preterm births at 24–31 and 32–26 weeks gestation (S2 and S3 Tables).

## Discussion

This study provides evidence that placental abnormalities may be associated with fetal growth abnormalities culminating in stillbirth or they may be associated with stillbirth without growth abnormalities. This conclusion is supported by the observation that 10 of the 11 placental findings associated with fetal growth abnormalities were also associated with higher risk of stillbirth. Another 5 placental findings were associated with stillbirth but not fetal growth abnormalities. Five patterns of association between placental findings, fetal growth abnormalities, and stillbirth were identified. Placental findings were associated with: (1) stillbirth but not fetal growth abnormalities; (2) fetal growth abnormalities in stillbirths only; (3) fetal growth abnormalities in live births only; (4) fetal growth abnormalities in stillbirths and live births in a similar manner; (5) a different pattern of fetal growth abnormalities in stillbirths and live births. These distinct patterns of association may indicate different mechanisms of placental function impairment and suggest different clinical management strategies.

### Mechanistic insights

Placental findings associated with stillbirth but not fetal growth abnormalities (group 1) may affect placental function acutely and severely. Such a sudden and severe impairment of placental function could lead to stillbirth without sufficient time necessary to affect fetal growth or through mechanisms unrelated to fetal growth. Acute chorioamnionitis of the placental membranes and chorionic plate, fetal chorionic plate vascular degenerative changes, perivillous or intervillous fibrin or fibrinoid depositions, and fetal vascular thrombi in the chorionic plate were findings of this type. It is unlikely that the five placental findings associated with stillbirth but not fetal growth could in some cases represent post-mortem changes. The changes the placental tissue undergoes in fetal death are well established and are unlikely to be confused with the underlying pathology. The only lesion that can continue for a limited time and to a very limited degree is acute chorioamnionitis of the placental membranes. There can be a mild polymorphonuclear response in the membranes after fetal death, especially in a case of severe amniotic fluid infection syndrome but that can be differentiated by other findings.

The second group of placental findings, consisting of velamentous umbilical cord insertion, terminal villous hypoplasia, and parenchymal infarction, was associated with fetal growth abnormalities among preterm stillbirths only (group 2). This suggests that when these findings impair placental function chronically and severely leading to fetal growth abnormalities stillbirth may result. Milder forms might not substantially affect fetal growth and may result in live birth rather than stillbirth.

A single umbilical artery was associated with fetal growth abnormalities in term live births only (group 3). This pattern of association suggests that single umbilical artery may impair placental function chronically and mildly leading to fetal growth impairment that becomes obvious late in pregnancy and without causing stillbirth.

Terminal villous hypoplasia and parenchymal infarction appear to have different effects in preterm and term pregnancies. In preterm pregnancies significant associations with fetal growth abnormalities were found in stillbirths only (group 2), while in term pregnancies significant associations with fetal growth abnormalities were found among live births only (group 3). This may suggest that a more severe form of terminal villous hypoplasia and parenchymal infarction can lead to fetal growth abnormalities culminating in stillbirth of the fetus before reaching term, while a less severe form of these placental findings leads to fetal growth abnormalities, but does not lead to stillbirth and results in fetal growth abnormalities reaching term. However, it should be noted that the relatively small (weighted) numbers of preterm live births and of term stillbirths in the fetal growth categories likely limited our ability to detect significant associations in these groups.

Similar relationships with fetal growth in stillbirths and live births (group 4) were observed for placental weight in both preterm stillbirths and live births and for intraparenchymal thrombus in term stillbirths and live births. The similar patterns of association suggest chronic impairment of placental function leading to fetal growth abnormalities in both stillbirths and live births. In pregnancies ending in stillbirth, this pattern suggests a second hit phenomenon in consequence of which the stillbirth occurs. The first hit may result in fetal growth abnormalities without resulting in stillbirth such that some placental findings are associated with fetal growth abnormalities in both live births and stillbirths. In some of the pregnancies with such induced growth abnormalities, a second insult may result in a stillbirth.

The last group includes placental findings with a different patterm of fetal growth in stillbirths and live births (group 5). This suggests that these placental findings impair placental function chronically affecting fetal growth. In pregnancies ending in stillbirth the placental findings may be more severe resulting in growth abnormalities incompatible with live birth. In live births the placental findings may be milder resulting in growth abnormalities but without stillbirth. In preterm pregnancies, the placental findings of this type were terminal villous immaturity, avascular villi, placental edema and retroplacental hematoma and in term pregnancies retroplacental hematoma, placental weight, and birth weight to placental weight ratio. In term pregnancies, placental weight was similar between SGA and AGA stillbirths and live births, but was substantially higher in LGA live births than in LGA stillbirths. The birth weight to placental weight ratio in term pregnancies was similar in all fetal growth groups among live births, but was substantially smaller in SGA stillbirths and larger in LGA stillbirths. Term SGA stillbirths had mean birth weight to placental weight ratio of 6.8, substantially lower than AGA and LGA term stillbirths (7.7 and 9.3, respectively). Thus birth weight was approximately 6, 7, and 9 times larger than placenta weight in the term stillbirth groups. Such an observation suggests absolute placental insufficiency in SGA stillbirths with placental size and function insufficient to support fetal growth and then life. In LGA stillbirths, the high birth weight to placental weight ratio indicates a relative placental insufficiency with placental size and function large but insufficient to support an even larger fetus [14].

Stillbirths and live births with congenital anomalies were included in the analysis as well as births resulting from pregnancies with complications. Although the proportion of babies with congenital anomalies was higher in stillbirths than in live births, as were the proportions born to mothers with pregnancy complications including chronic hypertension, gestational hypertension/pre-eclampsia, and pre-gestational diabetes, we have previously shown that the pattern of association observed between fetal growth and stillbirth in the complete cohort was similar to that in the subset excluding births with congenital anomalies and the subset without maternal diabetes or hypertension/pre-eclampsia [8]. Further, since pregnancy complications are very likely mediating factors in the relationship between fetal growth abnormalities and placental findings, it would not be appropriate to adjust for them in the multivariable analysis. Macerated stillbirths were also included. Although maceration may have made evaluation of birth weight more difficult, we previously found that the association between fetal growth and risk of stillbirth observed in the entire study population was also observed in the subset with optimal estimates of fetal age that included non-macerated stillbirths with a short interval of less than 7 days between the time they were last reported alive and first identified as demised [8]. Subset analyses of the association between fetal growth and placental findings were not undertaken here due to small sample sizes in some birth weight percentile categories and the corresponding risk of false negative findings. Although preterm pregnancies and their placentas, including AGA pregnancies, are not normal by definition, their inclusion allowed us to account for the effects of preterm gestational age while examining associations between placental findings and fetal growth abnormalities.

### Clinical implications

These distinct patterns of association between placental abnormalities, fetal growth, and stillbirth suggest not only different mechanisms of placental function impairment, but also different clinical management strategies. Some of the placental abnormalities, such as velamentous cord insertion, single umbilical artery, and placental volume can be detected during pregnancy using ultrasound [15, 16]. Placental volume correlates well with placental weight and is a good proxy for placental weight [17]. Other placental abnormalities, which are associated with stillbirth are at this time not detectable prenatally, but provide an important target of investigation within initiatives such as The Human Placenta Project [18]. The Human Placenta Project is a collaborative effort undertaken by the Eunice Kennedy Shriver National Institute of Child Health and Development “to understand the role of the placenta in health and disease”. It “aims to develop new tools to study the organ in real time to learn how it develops and functions throughout pregnancy. That knowledge may one day prevent and treat a range of common pregnancy complications, while providing insights into other areas of science and medicine as well.” https://www.nichd.nih.gov/research/HPP/Pages/default.aspx

The group of placental findings associated with stillbirth but not fetal growth abnormalities (group 1) appears to impair placental function in stillbirths severely and acutely and thus their diagnosis is at this time unlikely to affect clinical management in preterm pregnancies. This is because preterm delivery, which would prevent stillbirth, is associated with significant risk of neonatal mortality and morbidity that strongly depends on gestational age at delivery [19, 20]. In preterm pregnancies with these placental abnormalities, it would be difficult to decide when to intervene with delivery. However, presence of these placental abnormalities in term pregnancies could be a reasonable indication for delivery, if validated in future studies, since at term the risks of neonatal mortality and morbidity are very low and are likely outweighed by the risk of stillbirth [20–22].

In pregnancies with placental findings associated with fetal growth abnormalities in stillbirths only (group 2), and with placental findings associated with fetal growth abnormalities in both stillbirths and live births (group 4 and 5), the placental function appears to be affected chronically and severely in pregnancies ending in stillbirth. When growth abnormalities occur with these placental abnormalities, the risk of stillbirth may be substantially increased. Further studies are needed to quantify the risk of stillbirth in pregnancies with these placental findings and growth abnormalities to determine the gestational age at which delivery is associated with a net benefit of preventing stillbirth while minimizing the risk of neonatal mortality and morbidity. The net benefit in preterm pregnancies depends strongly on gestational age and the associated neonatal mortality and morbidity risk. However, at term, the risk of stillbirth associated with concurrent presence of these placental abnormalities and fetal growth abnormalities would likely outweigh the very low risk of neonatal mortality and morbidity [20–22]. Similarily, intervention in pregnancies with placental findings associated with fetal growth abnormalities in live births only (group 3) would require knowledge of the risk of adverse pregnancy outcomes, especially neonatal mortality and morbidity, associated with those findings. Also needed are studies of novel methods of non-invasive evaluation of placental structure and function, such as the ones conducted within The Human Placental Project, which would allow prenatal identification of these placental findings [18].

The clinical applicability at this stage is limited and likely restricted to birth weight to placental weight ratio and a few other placental findings identifiable by ultrasound examination. Recent developments in the estimation of placental weight hold the promise of clinical application but will have to be evaluated for clinical effectiveness.

### Strengths

There are several strengths of this study. The SCRN was a population-based study, which attempted to capture all stillbirths and a representative sample of live births with geographic, racial and ethnic diversity. The population-based design strengthens the external validity of the study findings and thus their generalizability. This is also one of the largest studies of stillbirths with over 500 fetal deaths and well characterized phenotype. Another strength of the study is the use of an algorithm developed to estimate gestational age of stillbirths at the time of death [11]. This allowed a more accurate estimation of gestational age of stillbirth than one based on age at delivery, which is critical in precise estimation of fetal growth and its abnormalities [8]. Another strength of the study is a standardized placental examination with central training for pathologists [10]. Standardization of the placental examination increases its objectivity and decreases the variability of the placental findings.

### Limitations

Despite the large number of stillbirths in the study, the numbers in individual fetal growth categories stratified into preterm and term pregnancies were relatively small, particularly those for term stillbirths. This may have resulted in insufficient power to detect more subtle differences in the patterns of association between placental abnormalities and fetal growth and stillbirth. Thus, interpretation of placental findings in the groups with smaller numbers of subjects requires caution. While estimation of GA in stillbirth based on time of death rather than time of birth significantly improves the accuracy of assessment of fetal growth [8], the exact time of death is not known and thus estimation of GA is not absolutely precise and is subject to some misclassification. Nevertheless, our prior study demonstrated that the algorithm used to estimate time of death in stillbirths performs well in estimation of GA and fetal growth in stillbirths [8, 11]. Pathologists could not be blinded to the origin of the placenta from stillbirths or live births due to the need to perform both clinical and research placental examinations. Chronic chorioamnionitis was not included in this analysis, because it is clinically significant only if it is associated with diffuse chronic villitis (of unknown etiology). There were very few cases of chronic chorioamnionitis with diffuse chronic villitis among the stillbirths in the cohort preventing analysis of the association with fetal growth. For these reasons, we did not examine chronic chorioamnionitis.

The clinical management implications are limited by the fact that many of the placental findings cannot be detected prenatally at this time. However, there is a large initiative underway within The Human Placenta Project to develop novel methods of prenatal evaluation of placental structure and function [18]. It is thus likely that more placental abnormalities associated with the risk of stillbirth and fetal growth will become amenable to diagnosis prenatally.

### Summary

Placental function supports fetal growth and development and its impairment may result in fetal growth abnormalities and stillbirth. We identified placental abnormalities which impair placental function as demonstrated by fetal growth abnormalities and / or stillbirth. All placental abnormalities associated with fetal growth aberrations, except for terminal villous hypoplasia, were also associated with stillbirth. This observation indicates that these placental abnormalities are important for placental function.

### Implications

The patterns of association between placental abnormalities, fetal growth, and stillbirth provide insights into the mechanism of placental function impairment and stillbirth. They also suggest implications for clinical care, especially for the placental findings amenable to prenatal diagnosis using ultrasound and associated with stillbirth in pregnancies at term. Clinical implications are especially clear for birth weight to placental weight ratio. The birth weight to placental weight ratio could be estimated prenatally using estimated fetal weight to placental volume ratio. Pregnancies with the abnormal ratio, at increased risk of stillbirth at term, may benefit from early term delivery, because of very low risk of neonatal mortality and morbidity at term.

## Supporting information

S1 Table Examples of recoding 0 cells. As noted in Statistical Analysis, the observation with the smallest analysis weight was recoded as having the placental finding in cases where no infants in a birth weight percentile group had the finding for the purpose of estimating the interaction p-value. Also as noted, the revised proportions and resulting p-values for association for stillbirths and live births separately are not reported. However, below are two examples to illustrate the effect of this recoding on the proportions and the subsequent interaction test. Note that the effect is generally more pronounced among stillbirths, which have analysis weights near 1, than among live births, many of whom have very small weights.
24+ weeks GA at death (stillibirths) or delivery (live births).
A. Single umbilical artery.
B. Velamentous insertion.
(DOCX)

S2 Table Placental findings in stillbirths and live births small, average, and large for gestational age 24–31 weeks^1/^. ^1/^ Birth weight percentiles for GA were determined using Hadlock ultrasound norms and GA at death (stillbirths) or delivery (live births) by the SCRN algorithm.
^2/^ Weighted percentages and other statistics are shown. For stillbirths, information was missing as follows (unweighted n): velamentous insertion, 1; furcate insertion, 1; circummarginate insertion, 7; circumvallate insertion, 7; terminal villous immaturity, 2; terminal villous hypoplasia, 3; any developmental disorder, 8; acute chorioamnionitis—chorionic plate, 2; acute umbilical cord arteritis, 2; acute umbilical cord phlebitis, 1; chorionic plate acute vasculitis, 7; chorionic plate vascular degenerative changes, 7; acute diffuse villitis, 1; chronic diffuse villitis, 2; any inflammatory disorder, 9; retroplacental hematoma, 4; parenchymal infarction, 3; intraparenchymal thrombus, 2; perivillous, intervillous fibrin, fibrinoid deposition, 9; any maternal circulatory disorder, 7; fetal vascular thrombi in the chorionic plate, 5; avascular villi, 2; edema, 4; any fetal circulatory disorder, 5; placental weight, 2; ratio birth weight/placental weight, 2.
For live births, information was missing as follows (unweighted n): velamentous insertion, 2; furcate insertion, 2; circummarginate insertion, 6; circumvallate insertion, 6; terminal villous hypoplasia, 2; any developmental disorder, 8; acute chorioamnionitis—placental membranes, 2; acute chorioamnionitis—chorionic plate, 1; acute umbilical cord arteritis, 4; acute diffuse villitis, 1; chronic diffuse villitis, 1; retroplacental hematoma, 1; perivillous, intervillous fibrin, fibrinoid deposition, 7; any maternal circulatory disorder, 4.
^3/^ P-value by the adjusted Wald F test for an association between a placental finding and birth weight percentile among stillbirths and live births separately. For each continuous measurement, the test is for mean difference in weighted ranks.
^4/^ P-value by the adjusted Wald F test for whether the association between a placental finding and birth weight percentile differs for stillbirths and live births. For each continuous measure, the test was based on weighted ranks.
(DOCX)

S3 Table Placental findings in stillbirths and live births small, average, and large for gestational age 32–36 weeks^1/^. ^1/^ Birth weight percentiles for GA were determined using Hadlock ultrasound norms and GA at death (stillbirths) or delivery (live births) by the SCRN algorithm.
^2/^ Weighted percentages and other statistics are shown. For stillbirths, information was missing as follows (unweighted n): single umbilical artery, 1; velamentous insertion, 1; furcate insertion, 2; circummarginate insertion, 1; circumvallate insertion, 1; terminal villous immaturity, 1; any developmental disorder, 4; acute funisitis, 1; acute umbilical cord arteritis, 3; acute umbilical cord phlebitis, 2; any inflammatory disorder, 1; perivillous, intervillous fibrin, fibrinoid deposition, 3; any maternal circulatory disorder, 1; edema, 2; any fetal circulatory disorder, 1; placental weight, 1; ratio birth weight/placental weight, 1.
For live births, information was missing as follows (unweighted n): velamentous insertion, 2; furcate insertion, 3; circummarginate insertion, 2; circumvallate insertion, 2; terminal villous immaturity, 1; terminal villous hypoplasia, 1; any developmental disorder, 5; acute chorioamnionitis—chorionic plate, 1; chorionic plate acute vasculitis, 1; chorionic plate vascular degenerative changes, 1; acute diffuse villitis, 1; chronic diffuse villitis, 1; any inflammatory disorder, 1 parenchymal infarction, 1; intraparenchymal thrombus, 1; perivillous, intervillous fibrin, fibrinoid deposition, 7; any maternal circulatory disorder, 6; fetal vascular thrombi in the chorionic plate, 1; avascular villi, 1; any fetal circulatory disorder, 1.
^3/^ P-value by the adjusted Wald F test for an association between a placental finding and birth weight percentile among stillbirths and live births separately. For each continuous measurement, the test is for mean difference in weighted ranks.
^4/^ P-value by the adjusted Wald F test for whether the association between a placental finding and birth weight percentile differs for stillbirths and live births. For each continuous measure, the test was based on weighted ranks.
(DOCX)

S4 Table STROBE checklist. **STROBE 2007 (v4) Statement—Checklist of items that should be included in reports of *case-control studies*** *Give information separately for cases and controls in case-control studies and, if applicable, for exposed and unexposed groups in cohort and cross-sectional studies.
**Note:** An Explanation and Elaboration article discusses each checklist item and gives methodological background and published examples of transparent reporting. The STROBE checklist is best used in conjunction with this article (freely available on the Web sites of PLoS Medicine at http://www.plosmedicine.org/, Annals of Internal Medicine at http://www.annals.org/, and Epidemiology at http://www.epidem.com/). Information on the STROBE Initiative is available at www.strobe-statement.org.
(DOCX)
