# Supplementary material for: Altered fetal growth, placental abnormalities, and stillbirth
Source: PLoS One. 2017 Aug 18;12(8):e0182874. doi: 10.1371/journal.pone.0182874 (PMC5562325; doi:10.1371/journal.pone.0182874)
Supplement: S1 Table — As noted in Statistical Analysis, the observation with the smallest analysis weight was recoded as having the placental finding in cases where no infants in a birth weight percentile group had the finding for the purpose of estimating the interaction p-value. Also as noted, the revised proportions and resulting p-values for association for stillbirths and live births separately are not reported. However, below are two examples to illustrate the effect of this recoding on the proportions and the subsequent interaction test. Note that the effect is generally more pronounced among stillbirths, which have analysis weights near 1, than among live births, many of whom have very small weights. 24+ weeks GA at death (stillibirths) or delivery (live births). A. Single umbilical artery. B. Velamentous insertion. (DOCX) [file pone.0182874.s001.docx]

A. Single umbilical artery

|  | SGA | AGA | LGA | P-value for association, SBs & LBs separately | P-value for interaction |
| --- | --- | --- | --- | --- | --- |
| BEFORE RECODING |  |  |  |  | NT |
| Stillbirths | 7.5 | 9.5 | 7.6 | 0.83 |  |
| Live births | 2.1 | 1.8 | 0.0 | <0.001 |  |
| AFTER RECODING |  |  |  |  | 0.02* |
| Stillbirths | 7.5 | 9.5 | 7.6 | 0.83 |  |
| Live births | 2.1 | 1.8 | 0.05 | 0.001 |  |

B. Velamentous insertion

|  | SGA | AGA | LGA | P-value for association, SBs & LBs separately | P-value for interaction |
| --- | --- | --- | --- | --- | --- |
| BEFORE RECODING |  |  |  |  | NT |
| Stillbirths | 5.0 | 3.7 | 0.0 | 0.01 |  |
| Live births | 1.3 | 1.1 | 2.2 | 0.76 |  |
| AFTER RECODING |  |  |  |  | 0.61* |
| Stillbirths | 5.0 | 3.7 | 2.2 | 0.63 |  |
| Live births | 1.3 | 1.1 | 2.2 | 0.76 |  |
