# Supplementary material for: Altered fetal growth, placental abnormalities, and stillbirth
Source: PLoS One. 2017 Aug 18;12(8):e0182874. doi: 10.1371/journal.pone.0182874 (PMC5562325; doi:10.1371/journal.pone.0182874)
Supplement: S2 Table — 1/ Birth weight percentiles for GA were determined using Hadlock ultrasound norms and GA at death (stillbirths) or delivery (live births) by the SCRN algorithm. 2/ Weighted percentages and other statistics are shown. For stillbirths, information was missing as follows (unweighted n): velamentous insertion, 1; furcate insertion, 1; circummarginate insertion, 7; circumvallate insertion, 7; terminal villous immaturity, 2; terminal villous hypoplasia, 3; any developmental disorder, 8; acute chorioamnionitis—chorionic plate, 2; acute umbilical cord arteritis, 2; acute umbilical cord phlebitis, 1; chorionic plate acute vasculitis, 7; chorionic plate vascular degenerative changes, 7; acute diffuse villitis, 1; chronic diffuse villitis, 2; any inflammatory disorder, 9; retroplacental hematoma, 4; parenchymal infarction, 3; intraparenchymal thrombus, 2; perivillous, intervillous fibrin, fibrinoid deposition, 9; any maternal circulatory disorder, 7; fetal vascular thrombi in the chorionic plate, 5; avascular villi, 2; edema, 4; any fetal circulatory disorder, 5; placental weight, 2; ratio birth weight/placental weight, 2. For live births, information was missing as follows (unweighted n): velamentous insertion, 2; furcate insertion, 2; circummarginate insertion, 6; circumvallate insertion, 6; terminal villous hypoplasia, 2; any developmental disorder, 8; acute chorioamnionitis—placental membranes, 2; acute chorioamnionitis—chorionic plate, 1; acute umbilical cord arteritis, 4; acute diffuse villitis, 1; chronic diffuse villitis, 1; retroplacental hematoma, 1; perivillous, intervillous fibrin, fibrinoid deposition, 7; any maternal circulatory disorder, 4. 3/ P-value by the adjusted Wald F test for an association between a placental finding and birth weight percentile among stillbirths and live births separately. For each continuous measurement, the test is for mean difference in weighted ranks. 4/ P-value by the adjusted Wald F test for whether the association between a placen [file pone.0182874.s002.docx]

| Characteristic, % or as shown ^2/^ | SGA | AGA | LGA | P-value for association, SBs & LBs separately ^3/^ | P-value for interaction ^4/^ |
| --- | --- | --- | --- | --- | --- |
| Unweighted number of stillbirths | 74 | 47 | 12 |  |  |
| Weighted number of stillbirths | 71 | 44 | 13 |  |  |
|  |  |  |  |  |  |
| Unweighted number of live births | 31 | 52 | 4 |  |  |
| Weighted number of live births | 6 | 8 | 0.3 |  |  |
|  |  |  |  |  |  |
| DEVELOPMENTAL DISORDERS |  |  |  |  |  |
| Umbilical cord |  |  |  |  |  |
| Single umbilical artery |  |  |  |  | NT |
| Stillbirths | 9.1 | 7.8 | 18.6 | 0.71 |  |
| Live births | 0.0 | 0.0 | 0.0 |  |  |
| Velamentous insertion |  |  |  |  | NT |
| Stillbirths | 1.8 | 5.8 | 0.0 | 0.19 |  |
| Live births | 11.4 | 0.6 | 0.0 | 0.26 |  |
| Furcate insertion |  |  |  |  | NT |
| Stillbirths | 1.6 | 0.0 | 0.0 | 0.60 |  |
| Live births | 0.0 | 0.0 | 0.0 |  |  |
| Placental membranes |  |  |  |  |  |
| Circummarginate insertion |  |  |  |  | NT |
| Stillbirths | 10.4 | 17.7 | 23.9 | 0.37 |  |
| Live births | 2.3 | 7.0 | 0.0 | 0.29 |  |
| Circumvallate insertion |  |  |  |  | NT |
| Stillbirths | 4.0 | 2.7 | 0.0 | 0.21 |  |
| Live births | 0.0 | 5.3 | 0.0 | 0.41 |  |
| Fetal villous capillaries |  |  |  |  |  |
| Terminal villous immaturity (diffuse) |  |  |  |  | NT |
| Stillbirths | 7.8 | 8.0 | 23.4 | 0.49 |  |
| Live births | 0.0 | 4.0 | 0.0 | 0.25 |  |
| Terminal villous hypoplasia (diffuse) |  |  |  |  | NT |
| Stillbirths | 8.1 | 0.0 | 0.0 | 0.08 |  |
| Live births | 16.3 | 0.9 | 0.0 | 0.23 |  |
| Any developmental disorder |  |  |  |  | NT |
| Stillbirths | 33.2 | 40.4 | 53.8 | 0.42 |  |
| Live births | 26.2 | 16.4 | 0.0 | 0.22 |  |
| INFLAMMATORY DISORDERS |  |  |  |  |  |
| Maternal inflammatory response |  |  |  |  |  |
| Acute chorioamnionitis--placental membranes |  |  |  |  | 0.03 |
| Stillbirths | 27.1 | 25.3 | 5.8 | 0.08 |  |
| Live births | 15.9 | 53.8 | 23.4 | 0.05 |  |
| Acute chorioamnionitis--chorionic plate |  |  |  |  | 0.10 |
| Stillbirths | 11.4 | 16.6 | 5.8 | 0.45 |  |
| Live births | 8.3 | 46.3 | 23.4 | 0.04 |  |
| Fetal inflammatory response |  |  |  |  |  |
| Acute funisitis |  |  |  |  | NT |
| Stillbirths | 3.5 | 5.3 | 0.0 | 0.16 |  |
| Live births | 10.4 | 21.4 | 23.4 | 0.37 |  |
| Acute umbilical cord arteritis (one or more arteries) |  |  |  |  | NT |
| Stillbirths | 0.0 | 0.0 | 0.0 |  |  |
| Live births | 6.0 | 14.6 | 23.4 | 0.33 |  |
| Acute umbilical cord phlebitis |  |  |  |  | NT |
| Stillbirths | 1.1 | 2.8 | 0.0 | 0.42 |  |
| Live births | 8.4 | 10.5 | 23.4 | 0.77 |  |
| Chorionic plate acute vasculitis |  |  |  |  | NT |
| Stillbirths | 1.2 | 2.9 | 0.0 | 0.42 |  |
| Live births | 7.6 | 19.0 | 23.4 | 0.26 |  |
| Chorionic plate vascular degenerative changes |  |  |  |  | NT |
| Stillbirths | 6.0 | 10.6 | 0.0 | 0.05 |  |
| Live births | 0.0 | 1.4 | 0.0 | 0.61 |  |
| Villitis |  |  |  |  |  |
| Acute diffuse villitis |  |  |  |  | NT |
| Stillbirths | 2.6 | 1.6 | 0.0 | 0.29 |  |
| Live births | 0.0 | 1.0 | 0.0 | 0.61 |  |
| Chronic diffuse villitis |  |  |  |  | NT |
| Stillbirths | 2.6 | 0.0 | 0.0 | 0.37 |  |
| Live births | 9.8 | 2.0 | 0.0 | 0.33 |  |
| Any inflammatory disorder |  |  |  |  | 0.06 |
| Stillbirths | 38.7 | 35.9 | 5.8 | 0.02 |  |
| Live births | 23.7 | 58.4 | 23.4 | 0.08 |  |
| CIRCULATORY DISORDERS |  |  |  |  |  |
| Maternal circulatory disorders |  |  |  |  |  |
| Retroplacental hematoma |  |  |  |  | NT |
| Stillbirths | 28.5 | 17.9 | 0.0 | 0.005 |  |
| Live births | 6.9 | 8.0 | 23.4 | 0.77 |  |
| Parenchymal infarction |  |  |  |  |  |
| Focal |  |  |  |  | NT |
| Stillbirths | 12.6 | 11.3 | 19.9 | 0.82 |  |
| Live births | 3.9 | 10.7 | 0.0 | 0.35 |  |
| Multifocal |  |  |  |  | NT |
| Stillbirths | 25.5 | 10.6 | 0.0 | 0.003 |  |
| Live births | 40.4 | 2.4 | 0.0 | 0.01 |  |
| Diffuse |  |  |  |  | NT |
| Stillbirths | 9.1 | 5.3 | 6.2 | 0.76 |  |
| Live births | 0.0 | 0.0 | 0.0 |  |  |
| Any parenchymal infarction |  |  |  |  | NT |
| Stillbirths | 47.2 | 27.2 | 26.1 | 0.07 |  |
| Live births | 44.3 | 13.1 | 0.0 | 0.03 |  |
| Intraparenchymal thrombus |  |  |  |  | 0.73 |
| Stillbirths | 16.1 | 26.9 | 26.0 | 0.35 |  |
| Live births | 22.8 | 20.4 | 23.4 | 0.99 |  |
| Perivillous, intervillous fibrin, fibrinoid deposition (diffuse) |  |  |  |  | 0.26 |
| Stillbirths | 9.1 | 12.9 | 9.3 | 0.84 |  |
| Live births | 7.3 | 1.4 | 10.2 | 0.37 |  |
| Any maternal circulatory disorder |  |  |  |  | 0.83 |
| Stillbirths | 64.0 | 62.3 | 55.4 | 0.87 |  |
| Live births | 56.3 | 42.6 | 33.6 | 0.65 |  |
| Fetal circulatory disorders |  |  |  |  |  |
| Fetal vascular thrombi in the chorionic plate |  |  |  |  | NT |
| Stillbirths | 23.8 | 34.0 | 27.4 | 0.54 |  |
| Live births | 4.9 | 14.6 | 0.0 | 0.23 |  |
| Avascular villi |  |  |  |  |  |
| Focal |  |  |  |  | NT |
| Stillbirths | 9.3 | 2.3 | 9.3 | 0.19 |  |
| Live births | 0.0 | 0.0 | 0.0 |  |  |
| Multifocal |  |  |  |  | NT |
| Stillbirths | 5.9 | 17.3 | 13.3 | 0.17 |  |
| Live births | 7.4 | 0.0 | 0.0 | 0.40 |  |
| Diffuse |  |  |  |  | NT |
| Stillbirths | 3.1 | 9.1 | 0.0 | 0.10 |  |
| Live births | 0.0 | 0.0 | 0.0 |  |  |
| Any avascular villi |  |  |  |  | NT |
| Stillbirths | 18.2 | 28.7 | 22.6 | 0.45 |  |
| Live births | 7.4 | 0.0 | 0.0 | 0.40 |  |
| Edema (placental hydrops) |  |  |  |  | NT |
| Stillbirths | 4.3 | 0.0 | 19.9 | 0.08 |  |
| Live births | 1.9 | 5.4 | 0.0 | 0.32 |  |
| Any fetal circulatory disorder |  |  |  |  | NT |
| Stillbirths | 40.7 | 51.2 | 58.9 | 0.38 |  |
| Live births | 11.7 | 20.0 | 0.0 | 0.21 |  |
| Placental weight |  |  |  |  | 0.07 |
| Stillbirths |  |  |  | <0.001 |  |
| Median (IQR) | 163  (117-193) | 224  (166-281) | 316  (159-545) |  |  |
| Live births |  |  |  | 0.05 |  |
| Median (IQR) | 191  (162-204) | 228  (185-263) | 258  (218-300) |  |  |
| Ratio birth weight/placental weight |  |  |  |  | 0.08 |
| Stillbirths |  |  |  | 0.53 |  |
| Median (IQR) | 4.6 (3.8-5.9) | 4.7 (3.7-6.1) | 5.0 (3.6-5.6) |  |  |
| Live births |  |  |  | 0.002 |  |
| Median (IQR) | 5.6 (4.9-7.2) | 5.0 (4.4-6.2) | 4.0 (3.4-4.6) |  |  |
