# Supplementary material for: Altered fetal growth, placental abnormalities, and stillbirth
Source: PLoS One. 2017 Aug 18;12(8):e0182874. doi: 10.1371/journal.pone.0182874 (PMC5562325; doi:10.1371/journal.pone.0182874)
Supplement: S3 Table — 1/ Birth weight percentiles for GA were determined using Hadlock ultrasound norms and GA at death (stillbirths) or delivery (live births) by the SCRN algorithm. 2/ Weighted percentages and other statistics are shown. For stillbirths, information was missing as follows (unweighted n): single umbilical artery, 1; velamentous insertion, 1; furcate insertion, 2; circummarginate insertion, 1; circumvallate insertion, 1; terminal villous immaturity, 1; any developmental disorder, 4; acute funisitis, 1; acute umbilical cord arteritis, 3; acute umbilical cord phlebitis, 2; any inflammatory disorder, 1; perivillous, intervillous fibrin, fibrinoid deposition, 3; any maternal circulatory disorder, 1; edema, 2; any fetal circulatory disorder, 1; placental weight, 1; ratio birth weight/placental weight, 1. For live births, information was missing as follows (unweighted n): velamentous insertion, 2; furcate insertion, 3; circummarginate insertion, 2; circumvallate insertion, 2; terminal villous immaturity, 1; terminal villous hypoplasia, 1; any developmental disorder, 5; acute chorioamnionitis—chorionic plate, 1; chorionic plate acute vasculitis, 1; chorionic plate vascular degenerative changes, 1; acute diffuse villitis, 1; chronic diffuse villitis, 1; any inflammatory disorder, 1 parenchymal infarction, 1; intraparenchymal thrombus, 1; perivillous, intervillous fibrin, fibrinoid deposition, 7; any maternal circulatory disorder, 6; fetal vascular thrombi in the chorionic plate, 1; avascular villi, 1; any fetal circulatory disorder, 1. 3/ P-value by the adjusted Wald F test for an association between a placental finding and birth weight percentile among stillbirths and live births separately. For each continuous measurement, the test is for mean difference in weighted ranks. 4/ P-value by the adjusted Wald F test for whether the association between a placental finding and birth weight percentile differs for stillbirths and live births. For each continuous measure, the test was ba [file pone.0182874.s003.docx]

| Characteristic, % or as shown ^2/^ | SGA | AGA | LGA | P-value for association, SBs & LBs separately ^3/^ | P-value for interaction ^4/^ |
| --- | --- | --- | --- | --- | --- |
| Unweighted number of stillbirths | 48 | 40 | 10 |  |  |
| Weighted number of stillbirths | 50 | 39 | 9 |  |  |
|  |  |  |  |  |  |
| Unweighted number of live births | 15 | 61 | 14 |  |  |
| Weighted number of live births | 17 | 50 | 12 |  |  |
|  |  |  |  |  |  |
| DEVELOPMENTAL DISORDERS |  |  |  |  |  |
| Umbilical cord |  |  |  |  |  |
| Single umbilical artery |  |  |  |  | NT |
| Stillbirths | 7.3 | 14.0 | 0.0 | 0.07 |  |
| Live births | 4.0 | 1.2 | 0.0 | 0.41 |  |
| Velamentous insertion |  |  |  |  | NT |
| Stillbirths | 10.2 | 3.4 | 0.0 | 0.11 |  |
| Live births | 0.0 | 2.7 | 0.0 | 0.60 |  |
| Furcate insertion |  |  |  |  | NT |
| Stillbirths | 1.8 | 3.1 | 0.0 | 0.42 |  |
| Live births | 0.0 | 2.5 | 8.9 | 0.30 |  |
| Placental membranes |  |  |  |  |  |
| Circummarginate insertion |  |  |  |  | 0.99 |
| Stillbirths | 11.0 | 12.0 | 19.7 | 0.81 |  |
| Live births | 5.6 | 7.4 | 12.4 | 0.80 |  |
| Circumvallate insertion |  |  |  |  | NT |
| Stillbirths | 0.0 | 3.2 | 8.8 | 0.36 |  |
| Live births | 0.0 | 1.0 | 0.0 | 0.61 |  |
| Fetal villous capillaries |  |  |  |  |  |
| Terminal villous immaturity (diffuse) |  |  |  |  | NT |
| Stillbirths | 6.6 | 8.2 | 14.3 | 0.84 |  |
| Live births | 9.7 | 1.4 | 0.0 | 0.28 |  |
| Terminal villous hypoplasia (diffuse) |  |  |  |  | NT |
| Stillbirths | 5.7 | 0.0 | 0.0 | 0.36 |  |
| Live births | 9.2 | 1.8 | 0.0 | 0.34 |  |
| Any developmental disorder |  |  |  |  | 0.78 |
| Stillbirths | 35.6 | 35.6 | 42.8 | 0.92 |  |
| Live births | 29.6 | 18.5 | 24.3 | 0.70 |  |
| INFLAMMATORY DISORDERS |  |  |  |  |  |
| Maternal inflammatory response |  |  |  |  |  |
| Acute chorioamnionitis--placental membranes |  |  |  |  | 0.76 |
| Stillbirths | 31.0 | 12.7 | 31.8 | 0.10 |  |
| Live births | 30.1 | 6.4 | 14.0 | 0.46 |  |
| Acute chorioamnionitis--chorionic plate |  |  |  |  | 0.83 |
| Stillbirths | 23.1 | 9.1 | 39.9 | 0.07 |  |
| Live births | 26.3 | 10.4 | 28.3 | 0.41 |  |
| Fetal inflammatory response |  |  |  |  |  |
| Acute funisitis |  |  |  |  | NT |
| Stillbirths | 1.5 | 2.8 | 0.0 | 0.43 |  |
| Live births | 0.0 | 1.4 | 0.0 | 0.61 |  |
| Acute umbilical cord arteritis (one or more arteries) |  |  |  |  | NT |
| Stillbirths | 0.0 | 0.0 | 12.0 | 0.60 |  |
| Live births | 0.0 | 1.4 | 3.4 | 0.40 |  |
| Acute umbilical cord phlebitis |  |  |  |  | NT |
| Stillbirths | 0.0 | 2.8 | 12.0 | 0.36 |  |
| Live births | 0.0 | 2.9 | 10.2 | 0.20 |  |
| Chorionic plate acute vasculitis |  |  |  |  | NT |
| Stillbirths | 2.2 | 2.8 | 8.1 | 0.77 |  |
| Live births | 0.0 | 6.9 | 0.0 | 0.18 |  |
| Chorionic plate vascular degenerative changes |  |  |  |  | NT |
| Stillbirths | 0.0 | 4.1 | 26.3 | 0.08 |  |
| Live births | 0.0 | 0.0 | 0.0 |  |  |
| Villitis |  |  |  |  |  |
| Acute diffuse villitis |  |  |  |  | NT |
| Stillbirths | 0.0 | 0.0 | 0.0 |  |  |
| Live births | 0.0 | 0.0 | 0.0 |  |  |
| Chronic diffuse villitis |  |  |  |  | NT |
| Stillbirths | 4.5 | 0.0 | 0.0 | 0.37 |  |
| Live births | 0.0 | 0.0 | 0.0 |  |  |
| Any inflammatory disorder |  |  |  |  | 0.76 |
| Stillbirths | 38.7 | 19.5 | 56.1 | 0.06 |  |
| Live births | 30.1 | 16.5 | 32.0 | 0.52 |  |
| CIRCULATORY DISORDERS |  |  |  |  |  |
| Maternal circulatory disorders |  |  |  |  |  |
| Retroplacental hematoma |  |  |  |  | NT |
| Stillbirths | 24.2 | 15.8 | 0.0 | 0.02 |  |
| Live births | 5.3 | 1.9 | 15.1 | 0.39 |  |
| Parenchymal infarction |  |  |  |  |  |
| Focal |  |  |  |  | NT |
| Stillbirths | 24.0 | 20.5 | 0.0 | 0.02 |  |
| Live births | 4.6 | 10.4 | 7.4 | 0.68 |  |
| Multifocal |  |  |  |  | NT |
| Stillbirths | 21.6 | 4.0 | 0.0 | 0.01 |  |
| Live births | 4.0 | 6.1 | 0.0 | 0.21 |  |
| Diffuse |  |  |  |  | NT |
| Stillbirths | 3.7 | 0.0 | 0.0 | 0.36 |  |
| Live births | 0.0 | 0.0 | 0.0 |  |  |
| Any parenchymal infarction |  |  |  |  | NT |
| Stillbirths | 49.4 | 24.5 | 0.0 | <0.001 |  |
| Live births | 8.6 | 16.6 | 7.4 | 0.51 |  |
| Intraparenchymal thrombus |  |  |  |  | 0.55 |
| Stillbirths | 20.5 | 19.0 | 24.6 | 0.94 |  |
| Live births | 11.5 | 9.8 | 34.9 | 0.31 |  |
| Perivillous, intervillous fibrin, fibrinoid deposition (diffuse) |  |  |  |  | NT |
| Stillbirths | 22.6 | 3.2 | 0.0 | 0.01 |  |
| Live births | 6.1 | 1.8 | 0.0 | 0.41 |  |
| Any maternal circulatory disorder |  |  |  |  | 0.09 |
| Stillbirths | 75.0 | 45.0 | 28.8 | 0.004 |  |
| Live births | 28.0 | 27.4 | 42.3 | 0.66 |  |
| Fetal circulatory disorders |  |  |  |  |  |
| Fetal vascular thrombi in the chorionic plate |  |  |  |  | 0.71 |
| Stillbirths | 19.2 | 19.2 | 41.4 | 0.47 |  |
| Live births | 10.1 | 8.6 | 9.1 | 0.98 |  |
| Avascular villi |  |  |  |  |  |
| Focal |  |  |  |  | NT |
| Stillbirths | 12.8 | 9.0 | 8.8 | 0.83 |  |
| Live births | 0.0 | 3.6 | 0.0 | 0.37 |  |
| Multifocal |  |  |  |  | NT |
| Stillbirths | 6.2 | 4.2 | 0.0 | 0.17 |  |
| Live births | 3.7 | 6.1 | 0.0 | 0.21 |  |
| Diffuse |  |  |  |  | NT |
| Stillbirths | 1.8 | 0.0 | 0.0 | 0.60 |  |
| Live births | 0.0 | 0.0 | 0.0 |  |  |
| Any avascular villi |  |  |  |  | NT |
| Stillbirths | 20.8 | 13.2 | 8.8 | 0.46 |  |
| Live births | 3.7 | 9.7 | 0.0 | 0.11 |  |
| Edema (placental hydrops) |  |  |  |  | NT |
| Stillbirths | 1.7 | 4.8 | 0.0 | 0.29 |  |
| Live births | 0.0 | 3.6 | 0.0 | 0.24 |  |
| Any fetal circulatory disorder |  |  |  |  | 0.63 |
| Stillbirths | 39.4 | 34.1 | 41.4 | 0.85 |  |
| Live births | 13.8 | 18.4 | 9.1 | 0.65 |  |
| Placental weight |  |  |  |  | 0.54 |
| Stillbirths |  |  |  | <0.001 |  |
| Median (IQR) | 239  (182-336) | 390  (306-430) | 428  (355-475) |  |  |
| Live births |  |  |  | <0.001 |  |
| Median (IQR) | 322  (295-338) | 376  (326-447) | 484  (403-573) |  |  |
| Ratio birth weight/placental weight |  |  |  |  | 0.43 |
| Stillbirths |  |  |  | 0.65 |  |
| Median (IQR) | 7.3 (5.6-8.4) | 6.9 (6.2-8.2) | 7.0 (6.6-9.1) |  |  |
| Live births |  |  |  | 0.24 |  |
| Median (IQR) | 6.1 (6.0-6.7) | 6.9 (5.9-8.0) | 6.5 (6.0-7.5) |  |  |
